# Outcome of Using Small-For-Size Grafts in Living Donor Liver Transplantation Recipients with High Model for End-Stage Liver Disease Scores: A Single Center Experience

**DOI:** 10.1371/journal.pone.0074081

**Published:** 2013-09-11

**Authors:** HongYu Li, Bo Li, YongGang Wei, LvNan Yan, TianFu Wen, MingQing Xu, WenTao Wang, JiaYin Yang

**Affiliations:** Department of Liver and Vascular Surgery, Center of Liver Transplantation, West China Hospital, Sichuan University, Chengdu, Sichuan Province, China; The University of Hong Kong, Hong Kong

## Abstract

**Aims:**

To evaluate the impact of small-for-size grafts (SFSG) in adult-to-adult living donor liver transplantation (AALDLT) on outcomes of recipients with different model for end-stage liver disease (MELD) score in a single liver transplant center.

**Materials and Methods:**

Clinical data of 118 patients underwent right-lobe AALDLT from January 2004 to December 2011 were retrospectively analyzed, Patients were divided into Group L (MELD score≤25) and Group H (MELD score > 25) according to MELD score. The patients were further stratiﬁed into Group LS (MELD score≤25, GBWR<0.8%), Group LN (MELD score≤25, GBWR≥0.8%), Group HS (MELD score > 25, GBWR<0.8%), and Group HN (MELD score > 25, GBWR≥0.8%) to investigate the impact of graft size on recipients’ complications and outcomes. Pre-operative characteristics, post-operative complications graded by the Clavien score and patient survival were analyzed.

**Results:**

MELD scores between the two groups were significant different (12.4±4.9 vs 34.5±7.5, P=0.026). There was no significant difference in preoperative demographic data as well as postoperative liver function. Complication rate, length of ICU and hospital stay, graft loss, and mortality were similar in both groups. The 1- and 3- year survival were similar between group H and group L. When recipients were further stratified into group LS, LN, HS, and HN, no significant difference was found among groups in 1- and 3- year survival rate. In multivariate analysis, HCC was not a predictor for long term survival.

**Conclusion:**

Our single institution experience demonstrates that it is safe to use SFSGs in high pre-MELD score recipients with the improvement of intensive care and the selection of listing criteria.

## Introduction

Adult to adult living donor liver transplantation (AALDLT) has become an established procedure as a partial solution for shortage of deceased donor graft [1]. The Model for End-stage Liver Disease (MELD) is a good tool to predict short-term mortality for patients on waiting list and has already been applied worldwide as an important variable for cadaveric graft allocation [2]. In 2002, the New York State Committee on Quality Improvement in Living Liver Donation prohibited live liver donation for potential recipients with MELD scores greater than 25 [3]. A partial liver graft transplanted into an adult recipient is often defined as a small-for-size graft [4]. It is generally accepted that such graft would be tolerate when the graft to body weight ratio (GBWR) is higher than 0.8%. However, GBWR<0.8% has been generally accepted as a predictor of small-for-size syndrome and subsequent irreversible graft damage which often needs a retransplantation [5]. In AALDLT, the graft size and pre-transplant MELD scores are important risk factors for patient post-transplant survival [6,7]. In this study, outcome of recipients in a single liver transplant center with different MELD scores using a SFSG in AALDLT recipients were evaluated and discussed.

## Patients and Methods

Data of 221 consecutive AALDLT recipients from January 2004 to December 2011 were retrospectively collected. Inclusion criteria were: 1) diagnosed with decompensated liver cirrhosis related to hepatitis B virus (HBV) or hepatocellular carcinoma (HCC) related to HBV; 2) right lobe grafts with or without middle hepatic vein (MHV). There were 118 recipients eventually enrolled in this study.

The operative procedures were similar to those performed in other major medical centers [8]. Preoperative international normalized ratios (INR), total bilirubin (TB) levels, and creatinine concentrations were used to calculate the MELD score and an additional point for HCC was not allotted. On the basis of preoperative MELD scores, patients were divided into two groups: Group L (MELD score≤25, n=102) and Group H (MELD score > 25, n=16). According to graft size, patients were stratified into 4 groups as follows: Group LS (MELD score≤25, GBWR<0.8%, n=23), Group LN (MELD score≤25, GBWR≥0.8%, n=79), Group HS (MELD score > 25, GBWR<0.8%, n=5), and Group HN (MELD score > 25, GBWR≥0.8%, n=11). The deﬁnitions used for complications were adapted from the Clavien grading system for negative outcomes [9]. Long-term outcomes were assessed by patient 1-, and 3- year survival rate.

### Ethics Statement

All clinical investigations were in accordance with the ethical guidelines of the Declaration of Helsinki. Ethical approval was obtained from the Committee of Ethics in West China Hospital of Sichuan University. Living donations were voluntary and altruistic in all cases, and written informed consent was obtained from both donors and recipients specifically to be involved in this study.

### Statistical analysis

Quantitative descriptive data were expressed as mean ± standard deviation (SD). Qualitative descriptive data were expressed as percentages. A chi-square test or Fisher’s exact test was used for categorical variables. Quantitative descriptive variables were analyzed by independent sample student t test. Patient survival between two groups was calculated with Kaplan-Meier survival analysis and compared with the log-rank tests. Statistical analyses were performed using SPSS version 16.0 (SSPS Inc, Chicago, Ill, USA). P values less than 0.05 were considered to be significant.

## Results

### Donors and recipients characteristics

MELD score between the two groups are significant different (12.4±4.9 vs 34.5±7.5, P=0.026). The preoperative characteristics of the donors, grafts, and recipients are summarized in Table 1. There were no significant difference in donor age (34.0±9.9 vs 37.8±8.0, P=0.122), donor gender (male: 67.6% vs 50.0%, P=0.168), donor BMI (23.4±2.5 vs 24.4±3.0, P=0.671), graft type (right lobe with MHV: 3.9% vs 6.3%, P=0.524), WIT (0.6±1.4 vs 1.3±2.2, P=0.081), CIT(2.6±5.4 vs 5.0±8.9, P=0.104), GBWR (1.23±0.27 vs 1.18±0.28, P=0.869), and operation time (414±87min vs 411±88min, P=0.851) between two groups.

**Table 1:** Donor and recipient characteristics for AALDLT.

	MELD≤25	MLED>25	P-value
Donor characteristics			
Donor age (years)	34.0±9.9	37.8±8.0	0.122(NS)
Donor gender (male)	67.6% (69/102)	50% (8/16)	0.168(NS)
Donor BMI	23.4±2.5	24.4±3.0	0.671(NS)
Cytotoxic antibody (positive)	0%(0/102)	0%(0/16)	-
Right with MHV	3.9% (4/102)	6.3% (1/16)	0.524(NS)
WIT(minutes)	46.1±5.1	53.5±6.8	0.073(NS)
CIT(minutes)	2.6±5.4	5.0±8.9	0.104(NS)
GBWR	1.23±0.27	1.18±0.28	0.869(NS)
Operation time (minutes)	414±87	411±88	0.851(NS)
Recipients characteristics			
Recipient age(years)	44.0±8.2	37.6±7.7	0.637(NS)
Recipient gender(male)	92.2% (94/102)	100% (16/16)	0.596(NS)
Recipient BMI	23.0±3.3	23.3±3.7	0.641(NS)
Recipient MELD	12.4±4.9	34.5±7.5	0.026
Causal pathology			
Decompensated cirrhosis	33.3% (34/102)	93.7% (15/16)	0.000
HCC	66.7% (68/102)	6.3% (1/16)	0.000
HbeAg-positive	18.6% (19/102)	25% (4/16)	0.550(NS)
HBV-DNA-positive	88.2 (90/102)	88.2 (90/102)	1.000(NS)
Child-Pugh grade			
A	38.2% (39/102)	0%(0/16)	0.001
B	48.0% (49/102)	12.5% (2/16)	0.013
C	13.7% (14/102)	87.5 (14/16)	0.000
Pretransplant complications			
Encephalopathy	1.0% (1/102)	6.3% (1/16)	0.254(NS)
Uncontrolled ascites	0%(0/102)	6.3% (1/16)	0.136(NS)
Peritonitis	0%(0/102)	0%(0/16)	-
Variceal bleeding	2.0% (2/102)	6.3% (1/16)	0.357(NS)
Waiting time to transplantation (days)	21.3±19.2	12.9±19.6	0.827(NS)
Operation time (minutes)	671.7±154.2	658.1±102.0	0.221(NS)

GBWR, graft to body weight ratio; BMI, body mass index; WIT, warm ischemia time; CIT, cold ischemia time; MELD, model for end-stage liver disease; HCC, hepatocellular carcinoma; MHV, middle hepatic vein

Recipient age (44.0±8.2 vs 37.6±7.7, P=0.637), recipient gender (male: 92.2% vs 100%, P=0.596), recipient BMI (23.0±3.3 vs 23.3±3.7, P=0.641), HbeAg-positive (18.6% vs 25%, P=0.550), HBV-DNA-positive (88.2% vs 93.7%, P=1.000), pretransplantation complications (encephalopathy: 1.0% vs 6.3%, P=0.254; uncontrolled ascites: 0% vs 6.3%, P=0.136; variceal bleeding: 2.0% vs 6.3%, P=0.357), waiting time to transplantation (21.3±19.2 days vs 12.9±19.6 days, P=0.827), and operation time (671.7±154.2min vs 658.1±102.0min, P=0.221) were similar in comparison of group L and H (Table 1). However, HBV-related HCC were more common in group L than in group H (66.7% vs 6.3%, P= 0.000). Child-Pugh grade A and B were more common in group L (A: 38.2% vs 0%, P=0.001; B: 48.0% vs 12.5%, P=0.013), but Child-Pugh grade C was more common in group H (C: 13.7% vs 87.5%, P=0.000).

### Postoperative complications and clinical outcome

Postoperative complications rates according to group L and H were similar (10.8% vs 6.3%, P=1.000). 11 recipients suffered postoperative complications in group L, including pneumonia (n=6), fluid collection (n=2), biliary complication (n=1), intraperitoneal bleeding (n=1), and hepatic artery thrombosis (n=1), while 1 recipient suffered intraperitoneal bleeding in group H. Grading the severity of the complications by Clavien score did not reveal a significant difference between two groups (P>0.05) as well as the length of ICU and hospital stay, graft loss, and mortality (P>0.05) (Table 2).

**Table 2:** Postoperative complications and clinical outcome of AALDLT recipients.

	MELD≤25	MLED>25	P-value
Complications (n)	10.8% (11/102)	6.3% (1/16)	1.000(NS)
Clavien score			
Grade I	6.9% (5/102)	0%(0/16)	1.000(NS)
Grade II	0%(0/102)	0%(0/16)	-
Grade III	3.9% (4/102)	6.3% (1/16)	0.524(NS)
Grade IV	0%(0/102)	0%(0/16)	-
Grade V	2.0% (2/102)	0%(0/16)	1.000(NS)
Length of ICU stay	11.5±8.3	13.8±10.4	0.098(NS)
Length of hospital stay	20.3±7.5	22.7±9.1	0.101(NS)
Graft loss			
Within hospital	2.0% (2/102)	0%(0/16)	1.000(NS)
Late	6.9% (7/102)	6.3% (1/16)	1.000(NS)
Mortality			
Within hospital	10.8% (11/102)	25% (4/16)	0.112(NS)
Late	25.5% (26/102)	37.5 (6/16)	0.315(NS)
Follow-up (months)	25.5% (26/102)	36.7±22.1	0.553(NS)

### Impact of MELD and GBWR on the patient survival

**Figure 1:**
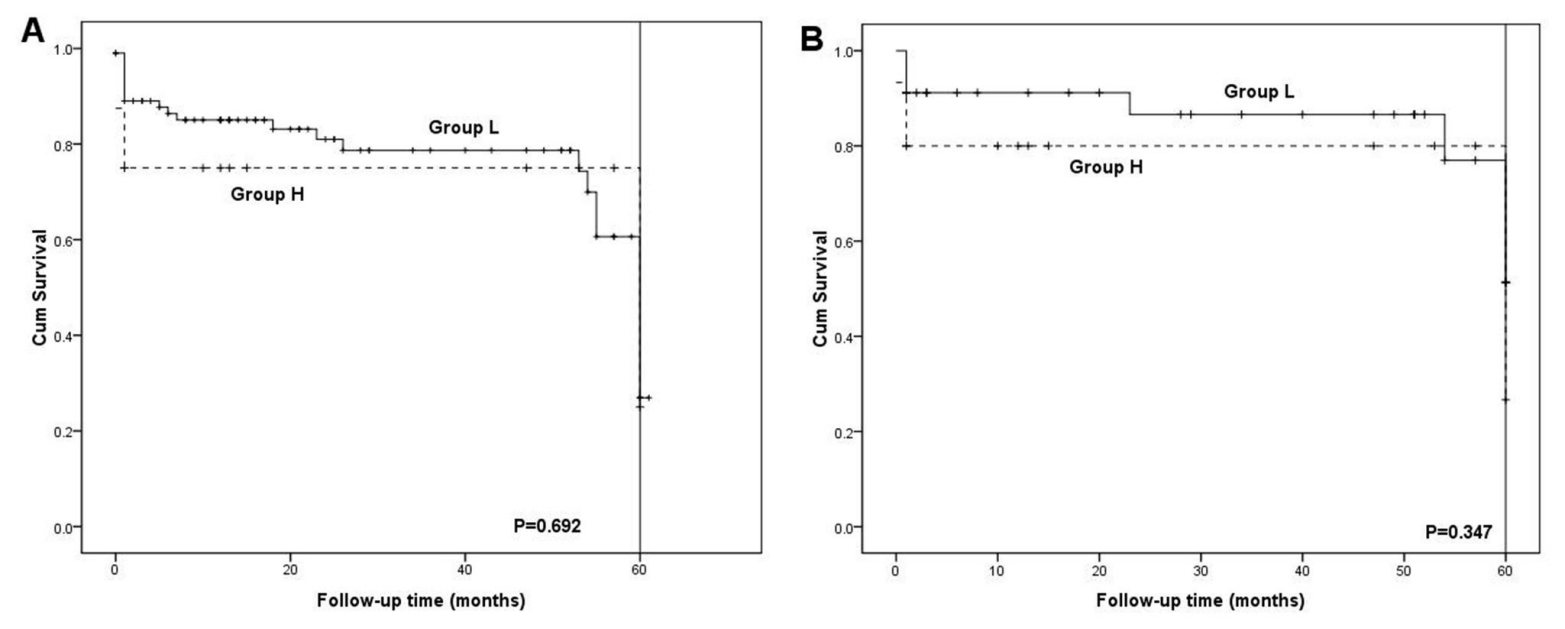
Comparison of the 1- and 3- year survival rates between group L and group H (A); Excluding HCC recipients, comparison of the 1- and 3- year survival rates between group L and group H (B).

**Figure 2:**
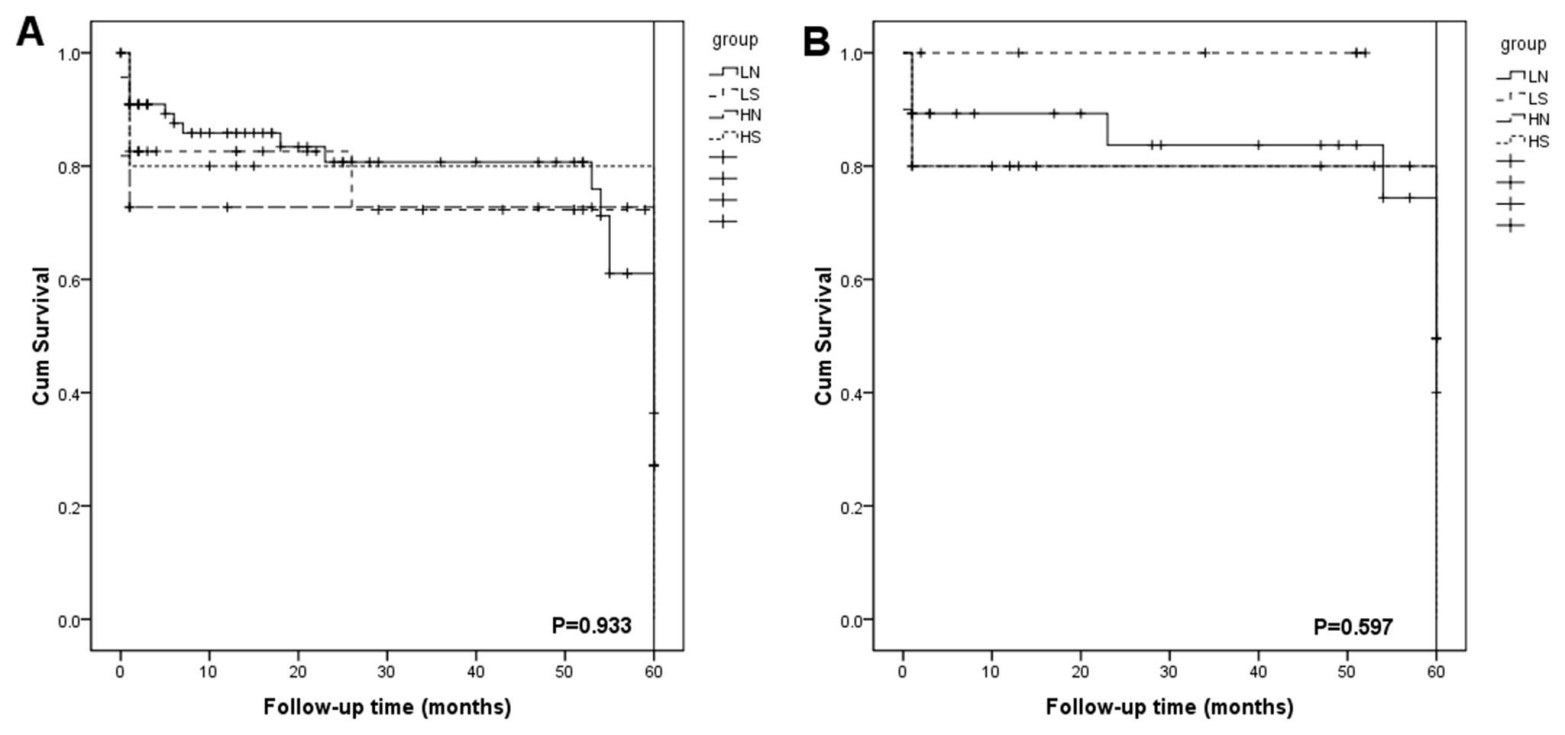
Comparison of the 1- and 3- year survival rates among group LS, LN, HS, and HN (A); Excluding HCC recipients, comparison of the 1- and 3- year survival rates among group LS, LN, HS, and HN (B).

The median follow-up time between two groups were similar (L: 36.7±22.1mo vs H: 32.3±19.9mo, P=0.553) (Table 2). The 1- and 3- year survival rates were 85.2% and 78.9% in group L, while 74% and 74% in group H (P=0.692). When recipients with HCC were excluded, the 1- and 3- year survival rates were 91.2% and 86.7% in group L and 80% and 80% in group H (P=0.347) (Figure 1). In the subgroup analysis based on both the MELD scores and graft size, The 1- and 3- year survival rates were 85.9% and 80.7% in group LN, 82.6% and 72.1% in group LS, 72.7% and 72.7% in group HN, and 80% and 80% in group HS (P=0.933). When recipients with HCC were excluded, the 1- and 3- year survival rates were 89.1% and 83.6% in group LN, 100% and 100% in group LS, 80% and 80% in group HN, and 79.7% and 79.7% in group HS (P=0.597) (Figure 2). Multivariate analysis revealed that accompanied HCC, GBWR, and MELD scores did not predict the 1- and 3- year survival rate (P>0.05) (Table 3).

**Table 3:** Risk Factors Multivariate Analysis.

Covariate	OR	95.0% C.I.	P-Value
Accompanied HCC	1.921	(0.671, 5.498)	0.599
GBWR	1.571	(0.355, 6.959)	0.553
MELD scores	1.039	(0.986, 1.095)	0.156

## Discussion

Since 2002, a continuous MELD severity score has been used in the USA to prioritize adult patients on the waiting list for liver transplantation [10]. However, debates still exist on the relationship between preoperative MELD score and postoperative clinical outcome. Theoretically, recipients with high MELD scores usually have worse preoperative conditions and experience a more complicated peri-operative course [11,12]. Hayashi et al. reported that there was no correlation between the 1-year survival rate and the preoperative MELD score [12]. Terrault et al. and Uchida et al. also reported that higher preoperative MELD score was a predictive factor for poor long-term survival [13,14]. According to Yi et al. report, recipients were stratiﬁed into a high score group (MELD score > 25) and a low score group (MELD score≤25) [15]. They reported that the 1-year survival rate among LDLT recipients without HCC to be similar between those two groups. And there was no signiﬁcant difference in the rate of postoperative complications. In our study, the postoperative complication rate after AALDLT did not differ between recipients with high versus low MELD scores. Pneumonia was still the first cause of death. The length of ICU and hospital stay was similar between the two groups. We attributed these to the improvements of peri-operative intensive care, including continued support of respiration and circulation, the adoption of effective anti-rejection therapy and powerful antibiotics, dynamic observation of bedside ultrasound for transplanted liver, and necessary adoption of artificial liver supporting system and dialysis treatment. Although using a high MELD score graft had a lower 1- and 3- year survival rate compared to a low MELD graft, there was no significant difference between them (P=0.692).

SFSG is defined as a partial liver graft transplanted into an adult recipient. When such a graft meets a critical size, it will be well tolerated. It is generally accepted that the GBWR should be higher than 0.8% to maintain a proper balance between liver regeneration and metabolic requirement [5]. The risk of Small-for-size syndrome and subsequent irreversible liver damage is relatively high when GBWR is less than 0.8%. Small-for-size syndrome often leads to graft dysfunction, which reduces the graft survival rate and increases the mortality rate. The pathogenesis of the syndrome is primarily related to graft volume, but recipients’ preoperative MELD score has been proved to contribute to its incidence [16]. Yi et al. also reported that a high MELD score (>25) did not predict the 1-year survival rate of HBV-infected recipients after ALDLT, and it was also not an important predictor of the 1-year survival rate in cases with an SFSG [15]. Reconstruction of the MHV or its major branches is well known as a solution to prevent SFSG and improve the postoperative clinical outcomes and survival rates. In our study, although 3.9% (4/102) in group L and 6.3% (1/16) in group H received right lobe with MHV, there was no significant difference among group LN, LS, HN, and HS (P>0.05). When a high MELD score recipient was operated, we might aggressively recommend reconstruction of the MHV in order to maintain the functional graft volume in those recipients with an SFSG.

Meantime, the diagnosis of hepatitis-B related HCC was another important predictor for the long term survival. Liver transplantation is an effective option in HCC patients who meet the Milan criteria [17]. In our study, the survival rates among groups with or without HCC were similar. We considered it might be the result of the adaption of rigorous listing criteria.

Although the small sample size and the retrospective design were a significant bias, our study demonstrated no significance in clinical outcomes and survival rates comparison between groups. Larger samples are needed to further corroborate our results in future. With the improvement of intensive care and the selection of listing criteria, we suggest that it is safe to use SFSGs in high pre-MELD score recipients.
